# The Mean Temperature of the Coldest Season is the Main Factor Influencing the Relationship Between Biodiversity and Biomass in the Desert Shrubland of Western Inner Mongolia

**DOI:** 10.1002/ece3.74353

**Published:** 2026-09-10

**Authors:** Zijing Li, Rui Jiao, Zhiyong Li, Cunzhu Liang, Tiejun Liu, Wentao Liang, Zhenhua Han, Kaixuan Li, Yanfei Zhang, Yaru Feng, Guoyong Wang

**Affiliations:** ^1^ Yinshanbeilu Grassland Eco‐Hydrology National Observation and Research Station China Institute of Water Resources and Hydropower Research Beijing People's Republic of China; ^2^ Institute of Pastoral Hydraulic Research, MWR Hohhot Inner Mongolia People's Republic of China; ^3^ Inner Mongolia Research Institute, China University of Mining and Technology (Beijing) Ordos People's Republic of China; ^4^ School of Ecology and Environment Inner Mongolia University Hohhot People's Republic of China; ^5^ Ministry of Education Key Laboratory of Ecology and Resource Use of the Mongolian Plateau & Inner Mongolia Key Laboratory of Grassland Ecology, School of Ecology and Environment Inner Mongolia University Hohhot Inner Mongolia People's Republic of China; ^6^ Collaborative Innovation Center for Grassland Ecological Security, Ministry of Education of China and Inner Mongolia Autonomous Region Inner Mongolia University Hohhot Inner Mongolia People's Republic of China

**Keywords:** aridity, community biomass, soil physicochemical properties, species richness, xerophytic shrub

## Abstract

The biodiversity‐ecosystem functioning (BEF) relationship has been a central focus in ecology, yet its nature and strength remain contentious. In natural ecosystems, these relationships are modulated by a suite of abiotic (e.g., climate, soil) and biotic (e.g., community composition) factors, and often result in complex and context‐dependent patterns. This study investigated xerophytic shrub ecosystems in western Inner Mongolia. We used Random Forest models to identify the key environmental drivers of biodiversity and community biomass. Based on these findings, Structural Equation Modeling was applied to disentangle the direct and indirect effects of environmental factors on biodiversity, biomass, and their relationship. Results showed that climatic factors are the primary drivers of community biomass, with the mean temperature of the coldest season being the key limiting climatic factor. Shrub species richness was primarily influenced by shrub density, followed by soil total carbon content. Species richness showed no significant direct effect on biomass. Instead, the mean temperature of the coldest season and soil pH directly determined shrub biomass, thereby modulating the observed BEF relationship. These findings extend BEF theory to natural ecosystems and underscore the role of winter temperature as a key driver of community biomass, with implications for predicting ecosystem responses to climate change.

## Graphical abstract

Shrub biomass was directly determined by mean winter temperature and soil pH, whereas species richness had no significant direct effect. Climatic factors modulated the biodiversity–ecosystem functioning relationship indirectly through biomass, underscoring winter temperature as a critical driver for predicting ecosystem responses to climate change.

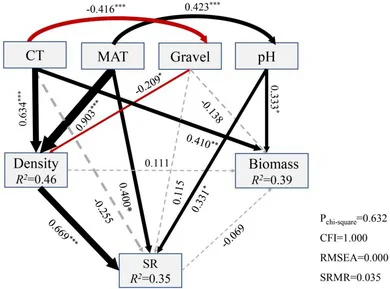

## 1 Introduction

The relationship between biodiversity and ecosystem functioning (BEF) has long been a central and highly active area of research in ecology (Cadotte and Tatsumi 2025; Cardinale et al. 2012; Gonzalez et al. 2020; Hooper et al. 2005; Tilman et al. 1996). In recent years, anthropogenic activities—including fossil fuel combustion, pollutant emissions, and land‐use change—have profoundly transformed terrestrial ecosystems (Steffen et al. 2015), triggering global warming and driving species loss at an unprecedented rate (Isbell et al. 2017; Pimm et al. 2014). Such rapid biodiversity loss compromises ecosystem functions and services (Dib et al. 2020), ultimately threatening the goods and services upon which human societies depend (Cardinale et al. 2012; Hooper et al. 2012; Isbell et al. 2013). Consequently, elucidating the BEF relationship is critical for understanding the ecological consequences of biodiversity loss and for informing the conservation and restoration of natural ecosystems.

Ecosystem functioning encompasses the suite of ecological processes—such as primary productivity, nutrient cycling, and decomposition—that regulate the fluxes of energy, nutrients, and organic matter within an environment (Cardinale et al. 2012). Among these processes, primary productivity is of fundamental importance, as it underpins virtually all ecosystem services and products (Duffy et al. 2017; Srivastava and Vellend 2005). Numerous controlled experiments have demonstrated that biodiversity loss is a major driver of declines in key ecosystem functions like productivity, with a broad consensus emerging from this work (Cardinale et al. 2011; Hector et al. 1999; Tilman et al. 2001). In fact, its impact can be comparable to, or even exceed, that of other major anthropogenic stressors or environmental changes (Duffy et al. 2017; Hautier et al. 2015; Oehri et al. 2017). Two major hypotheses have been proposed to explain how biodiversity influences ecosystem functioning. The niche complementarity hypothesis suggests that greater species richness enhances ecosystem functioning through complementary resource use among coexisting species (Tilman 1997). In contrast, the mass‐ratio hypothesis, originally proposed by Grime (Grime 1998), emphasizes that ecosystem processes are determined overwhelmingly by the functional traits of the dominant species in a community, and are relatively insensitive to the richness of less abundant species. Under this hypothesis, the effect of a species on ecosystem functioning is proportional to its relative abundance in the community. These two hypotheses are not mutually exclusive, and their relative importance may vary across ecosystems and environmental contexts.

However, findings from highly controlled experiments often conflict with observations from large‐scale natural ecosystems. In nature, the BEF relationship is more complex, exhibiting positive, negative, or non‐significant correlations. Consequently, there is intense debate about the applicability of experimental mechanisms and interpretations to natural contexts (Pillai and Gouhier 2019; Van der Plas 2019; Wardle 2016) This discrepancy highlights the need to further investigate the strength and direction of BEF relationships in natural systems. The BEF relationship is further modulated by environmental factors. Factors such as drought intensity (Li et al. 2021), precipitation variability (Li et al. 2024), and nutrient addition (Bondaruk et al. 2025) can directly regulate plant growth and development and, by altering plant diversity, profoundly influence community biomass. However, empirical research on this interplay has primarily focused on grassland ecosystems. For shrub communities, biodiversity is also a key biomass driver (Chen et al. 2025), and shrub biomass is highly sensitive to environmental changes like drought (Smith et al. 2024) and precipitation (Li et al. 2024). Yet, due to greater challenges in field biomass measurement (e.g., difficulties in destructive sampling), mechanistic studies on how environmental factors regulate the species richness‐shrub biomass relationship remain relatively scarce.

Shrubs and semi‐shrubs are important layers in the grassland and desert ecosystems of the central and western Mongolian Plateau. They support the activities of numerous wild animals and are crucial for maintaining the region's biodiversity and ecosystem stability (Richardson et al. 2014). Additionally, they play a key role in preventing soil erosion, windbreak, and sand fixation, and soil improvement. Therefore, elucidating the mechanisms through which environmental factors influence the diversity‐biomass relationship in shrub communities is crucial for accurately predicting and responding to changes in ecosystem functions under climate change.

This study examines how plant diversity and environmental factors jointly influence biomass of xerophytic shrub and semi‐shrub ecosystems in central and western Inner Mongolia. Random Forest models were employed to identify key environmental drivers affecting xerophytic shrub biodiversity and biomass. Furthermore, Structural Equation Modeling was applied to analyze how environmental factors influence biodiversity, community biomass, and their interrelationship. This study addresses two questions: (1) What are the main environmental factors influencing biodiversity and biomass in xeric shrub ecosystems? (2) How do environmental factors mediate the relationship between biodiversity and biomass in these ecosystems? Based on our observational data, we further discuss potential mechanisms that may explain the observed patterns. Ultimately, this work aims to deepen the understanding of the BEF relationship in xerophytic shrub ecosystems and to provide both data and theoretical support for their conservation and management.

## 2 Materials and Methods

### 2.1 Study Area and Sites Sampled

**FIGURE 1:**
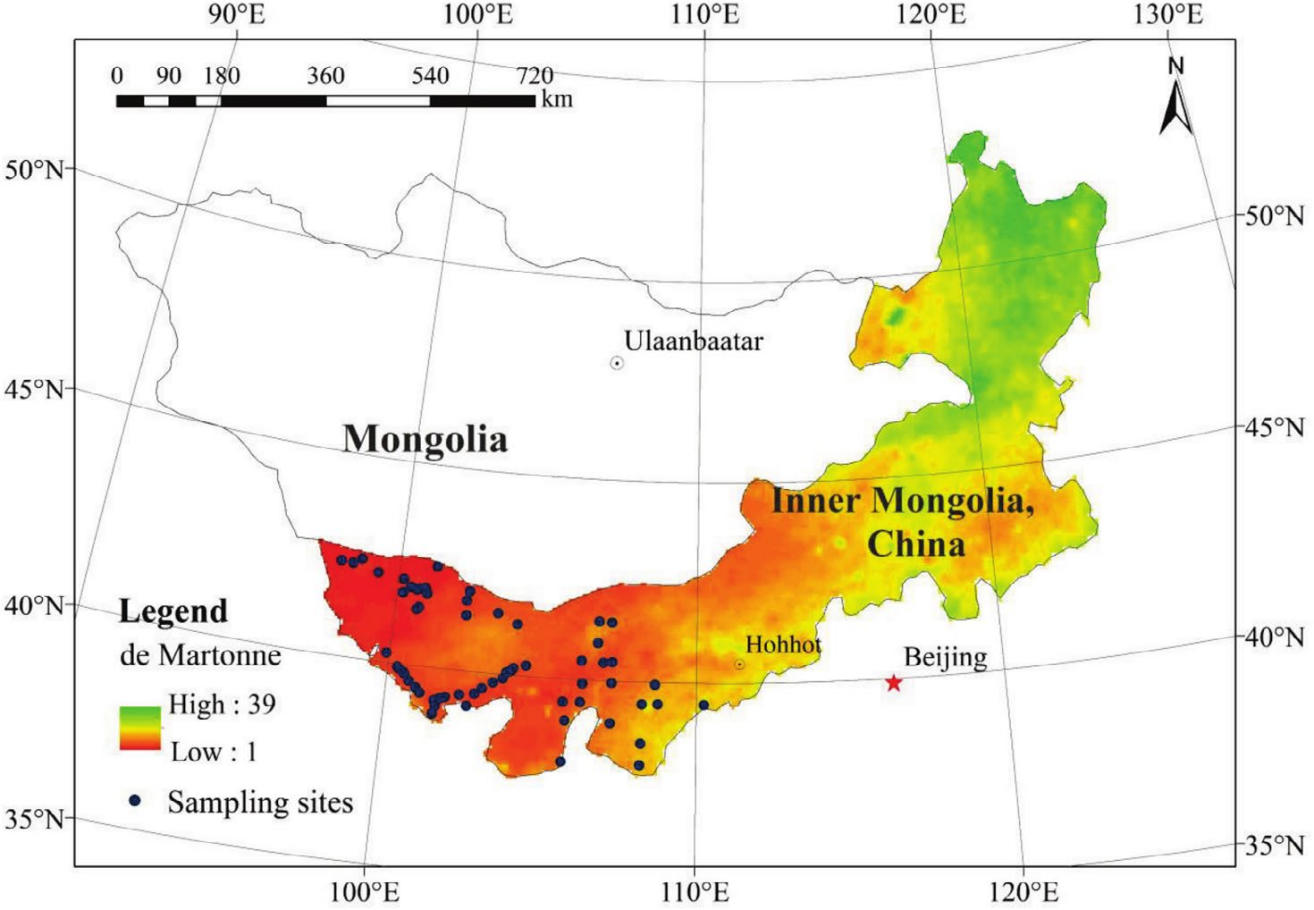
Location of the 61 sampling sites in the central and western Inner Mongolia, China.

This study was conducted in the central and western regions of Inner Mongolia, with the Alxa‐Ordos Plateau as the core area, encompassing primarily the Alxa League, Ordos City, and the Bayannur region (Figure 1). The investigation focused on shrub and semi‐shrub communities in desert and sandy land ecosystems. The geographic scope spanned from 37.93° to 42.50° N and 98.03° to 112.75° E, with elevations ranging between 826.0 and 1807.5 m. The regional climate is characterized by a mean annual temperature of 3.6°C–10.8°C and an average annual precipitation of 37–409 mm, with most rainfall occurring between June and August. The aridity index ranged from 1.86 to 23.24. Predominant soil types include gray‐brown desert soil and desert‐steppe brown calcareous soil.

Shrub community surveys were carried out during the growing season (July to September) from 2011 to 2018. A total of 61 survey plots were established across the study area (Figure 1). Sampling sites preferentially were located on flat terrain to minimize topographic effects. Areas under extreme conditions, such as those with high soil salinity or severe degradation, were avoided. Sites were selected in areas with relatively uniform species composition, community structure, and habitat conditions, and with minimal anthropogenic disturbance, in order to emphasize natural variation in biodiversity and its environmental drivers.

At each sampling site, three 10 m × 10 m quadrats were established, with a distance of approximately 50–100 m between adjacent quadrats. For each sampling site, basic geographic and environmental information was recorded, including latitude, longitude, elevation, habitat type, slope aspect, and slope gradient.

### 2.2 Data Collection

#### 2.2.1 Vegetation

At each of the three 10 m × 10 m quadrats per site, all shrub and semi‐shrub individuals were identified to species level, counted, and measured for crown dimensions and height. For each quadrat, we recorded: (1) Species identity and abundance of all shrub and semi‐shrub species; (2) For each individual: long‐axis crown diameter (L1), short‐axis crown diameter (L2), and maximum height (H); (3) Overall community cover and dominant species. Adjacent to the quadrat, dominant species of the shrub community were selected, and 3–5 undamaged, healthy plants—representing large, medium, and small crown‐size classes—were chosen as standard specimens for whole‐plant harvesting. Whole plants were excavated carefully to include the complete root system. Aboveground parts were separated into old branches, current‐year branches, and leaves. Roots were washed to remove soil and debris. All components were weighed fresh in the field, placed in labeled cloth bags, transported to the laboratory, and oven‐dried at 65°C to constant weight (typically 72–96 h). Dry weights were recorded to 0.01 g precision. For each species, a minimum of 15 individuals (across all sites) were harvested to develop species‐specific allometric equations.

#### 2.2.2 Soil

Soil bulk density was determined from soil profiles. At each site, a representative area adjacent to the quadrats was selected, and a soil profile (1.5 m × 0.8 m × 1 m, L × W × D) was excavated perpendicular to the ground surface, continuing to the parent material in cases of shallow soil. Using a 100 cm^3^ cutting ring, bulk density samples were collected from seven depth intervals: 0–5, 5–10, 10–20, 20–30, 30–50, 50–70, and 70–100 cm. Samples were sealed in plastic bags for transport.

For other soil properties, composite samples were obtained using a soil auger. Within each large quadrat, three soil cores were collected along a diagonal line at the same seven depth intervals. Soil from the same depth across the three cores was thoroughly mixed into a single composite sample per depth, which was then sealed and preserved. This yielded three composite samples per depth per site for subsequent analysis of soil physical and chemical properties.

In the laboratory, visible roots and stones were removed from all samples. Soil was air‐dried at room temperature and sieved through a 2‐mm mesh for analysis. Soil bulk density was calculated as the ratio of the oven‐dry weight (105°C to constant weight) to the volume of the cutting ring. The soil‐rock ratio was determined as the weight of gravel (> 2 mm) divided by the total weight of the air‐dried sample. Soil pH was measured potentiometrically (soil: water ratio of 1:2.5) after a 30‐min equilibration period using a pH meter (Mettler Toledo, Shanghai). Soil total carbon (STC) and total nitrogen (STN) were analyzed with an elemental analyzer (VARIO EL III, Elementary, Germany) at the Institute of Botany, Chinese Academy of Sciences. Soil total phosphorus (STP) was determined by the molybdate colorimetric method following H_2_SO_4_‐H_2_O_2_ digestion. The resulting soil attribute data are summarized in Table A1.

#### 2.2.3 Climate Data

Climate data were sourced from the CHELSA database (Climatologies at High Resolution for the Earth's Land Surface Areas, http://chelsa‐climate.org/). This dataset provides interpolated, long‐term average (1979–2013) climate variables at a spatial resolution of 30 arc sec. Key variables extracted included: mean annual temperature (MAT, °C), mean annual precipitation (MAP, mm), mean temperature of the coldest season (CT, °C), mean temperature of the warmest season (WT, °C), precipitation of the coldest season (CP, mm), and precipitation of the warmest season (WP, mm). Using ArcGIS 10.2.2, values for these variables were extracted for each sampling site based on its geographic coordinates. Summary statistics are provided in Table A1.

To characterize climatic moisture conditions across the study area, we employed the de Martonne aridity index (*I*_*dM*_) as a bioclimatic proxy for dryness/wetness (Tuhkanen 1980). This index is well‐suited for large‐scale studies and effectively captures vegetation‐climate relationships (Meng et al. 2004). The *I*_*dM*_ is calculated as MAP/(MAT + 10), where MAP is mean annual precipitation (mm) and MAT is mean annual temperature (°C). Higher index values indicate lower aridity (i.e., greater humidity).

### 2.3 Diversity and Biomass Assessment

#### 2.3.1 Shrub Species Richness

Shrub biodiversity was quantified using species richness. For each sampling site, species richness was recorded as the average number of species across the three replicate quadrats (units: species/100 m^2^; see Table A2).

#### 2.3.2 Shrub Density

Shrub density was calculated at the site level as the number of individual shrubs and semi‐shrubs per unit area. Specifically, it was determined by first counting all individuals within each of the three quadrats, then averaging the three quadrat‐level values (Table A2).

#### 2.3.3 Biomass

Individual plant biomass was estimated based on three morphological measurements: the major crown diameter (*L*_1_), minor crown diameter (*L*_2_), and plant height (*H*). Of the 31 shrub and semi‐shrub species surveyed, biomass for 20 species was calculated using existing allometric models from the *Handbook of Biomass Models for Common Shrubs in China* (Xie et al. 2018) and related studies (Dang et al. 2016; Zhao et al. 2019). For the remaining 11 species—*Artemisia blepharolepis*, *Convolvulus tragacanthoides*, *Caragana brachypoda*, *Gymnocarpos przewalskii* , *Brachanthemum pulvinatum*, *Oxytropis aciphylla*, *Nitraria sphaerocarpa*, *Calligonum mongolicum*, *Salsola laricifolia*, *Kalidium foliatum*, and *Salsola passerina*—site‐specific allometric equations were developed using harvested standard individuals (see Table A3 for details). During model construction, mean crown diameter (*D*), crown projection area (*A*), or crown projection volume (*V*) served as the independent variable(s) for fitting aboveground biomass (*AGB*) models. Belowground biomass (*BGB*) models were similarly fitted using these variables or the aboveground dry mass (*Ma*). Each independent variable was calculated as follows:

(1) $$D=\left(L_{1}+L_{2}\right)/2$$

(2) $$A=\pi \;\left(L_{1}\times L_{2}\right)/2$$

(3) V=A×H

The power function model was preferentially selected for biomass fitting, expressed as follows:

(4) $$y=a\times x^{b}$$

The expression can be transformed into a general linear model after taking natural logarithms, as follows:

(5) lny=lna+b×lnx

If the power function model showed inadequate goodness‐of‐fit, a general linear model was employed:

(6) y=ax+b

In Equations ((4), (5), (6)): *y* denotes the response variable (either belowground or aboveground biomass), *x* represents the predictor, specifically one of the following: mean crown diameter, crown projection area, crown projection volume, or aboveground dry matter. Parameters *a* and *b* are the fitted parameters.

Model goodness‐of‐fit was assessed using the significance test *p* value, the adjusted coefficient of determination (*R* ^2^_adj_), and the standard error of the estimate (SEE). For each species, the model with the highest *R* ^2^_adj_, the smallest SEE, and statistical significance (*p* < 0.05) was selected to describe the relationship between biomass (above‐ or below‐ground) and the predictor variable.

Total shrub biomass for a quadrat was calculated as the sum of the above‐ and below‐ground biomass of all plants within it. Community‐level biomass (kg/100 m^2^) for each sampling point was then derived by averaging the total biomass across its three replicate quadrats. All allometric growth equations were fitted using ordinary least squares (OLS) regression in *R*.

### 2.4 Statistical Analysis

First, a Random Forest model was implemented to identify environmental factors significantly influencing shrub community biomass and species richness (SR). Predictors included climatic variables (MAT, MAP, CT, WT, CP, WP, *I*_*dM*_), soil properties (pH, bulk density [BD], soil‐to‐stone ratio, STC, STN, STP), and shrub density. The model was constructed with 5000 decision trees, and the number of variables randomly sampled at each split was set to 5. Analyses were performed in R using the “*randomForest*” package (Liaw and Wiener 2002). Variable significance was assessed with the “*rfPermute*” package (Archer 2016), and model performance was evaluated via cross‐validated *R* ^*2*^ and overall model significance using the “*A3*” package (Fortmannroe 2015).

Second, to elucidate how environmental drivers mediate the relationship between species richness and community biomass, Structural Equation Modeling (SEM) was conducted. The predictors included in the SEM were restricted to the two climate and two soil factors that showed significant effects on total biomass in the Random Forest analysis. An initial full model incorporating all plausible pathways was specified, then refined based on goodness‐of‐fit criteria: a non‐significant chi‐square test (*p* > 0.05), standardized root mean square residual (SRMR < 0.10), and root mean square error of approximation (RMSEA < 0.05). Models meeting these thresholds were retained for interpretation. SEM was performed using the “*lavaan*” package (Rosseel 2012).

Prior to analysis, several variables were transformed to meet normality assumptions: natural log transformations were applied to total shrub biomass, MAP, WP, *I*_*dM*_, STC, pH, STP, and BD, while a square‐root transformation was used for the soil‐to‐stone ratio. All statistical computations were conducted in R x64 version 4.2.1 (R Development Core Team).

## 3 Results

### 3.1 Fitting of Shrub Biomass Equation Models

**FIGURE 2:**
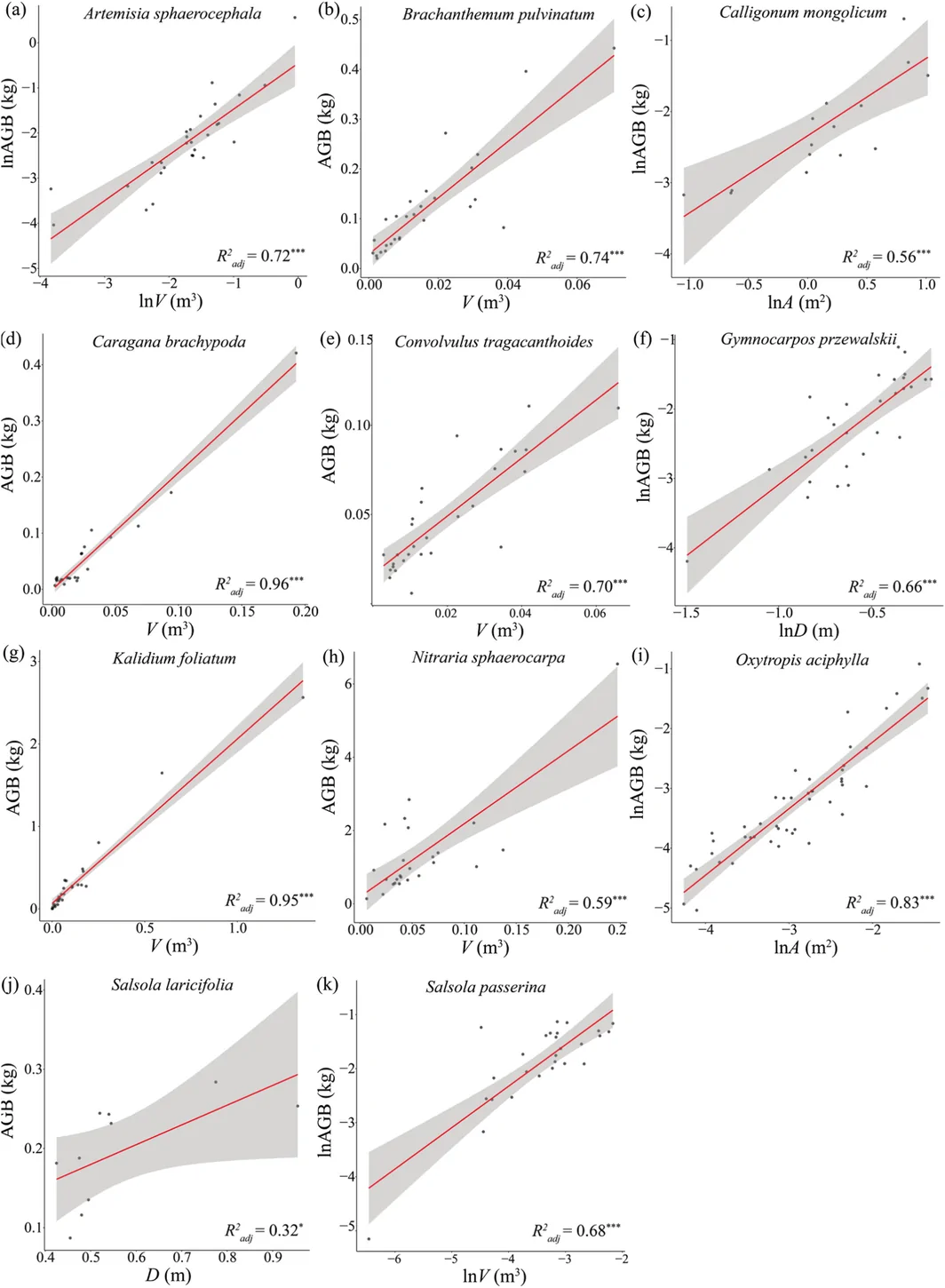
Best‐fit allometric equations for aboveground biomass of xerophytic shrub species. The red solid line indicates a significant fitted relationship; shaded area shows the 95% confidence interval. *D*, Mean crown diameter; *A*, Crown projection area; *V*, Crown projection volume; AGB, Aboveground biomass. Significance levels: *p < 0.05 and ***p < 0.001.

**FIGURE 3:**
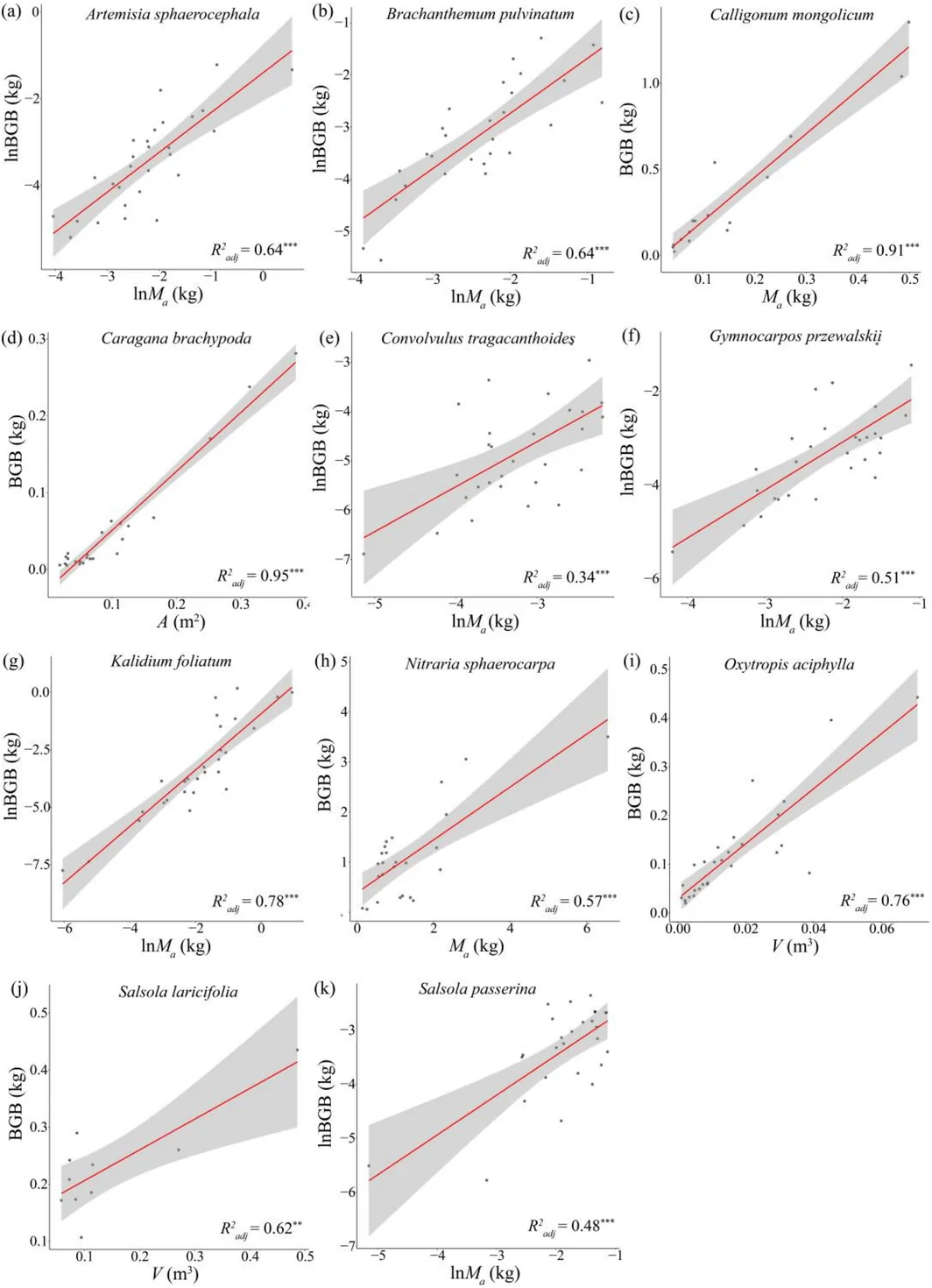
Best‐fit allometric equations for belowground biomass of xerophytic shrub species The red solid line indicates a significant fitted relationship; shaded area shows the 95% confidence interval. *A*, Crown projection area; *V*, Crown projection volume; *M*_*a*_, Aboveground dry matter; BGB, Belowground biomass. Significance levels: **p < 0.01 and ***p < 0.001.

The aboveground biomass (AGB) and belowground biomass (BGB) of shrubs were modeled using crown diameter, crown projection area, and crown projection volume, respectively. The results demonstrated that the allometric equations for biomass were well fitted across all species. With the exception of *Salsola laricifolia* (*p* < 0.05), the fits for both AGB and BGB of the remaining species reached a highly significant level (*p* < 0.001; Figures 2, 3). The adjusted coefficient of determination (*R* ^*2*^_*adj*_) for AGB ranged from 0.32 to 0.96 (Figure 2), while for BGB it ranged from 0.34 to 0.95 (Figure 3).

Among the best‐fit equations for AGB, crown projection volume served as the optimal predictor for 7 of the 11 species, whereas crown projection area and mean crown diameter each served as the best‐fitting variable for 2 species (Figure 2). For BGB, except for *Oxytropis aciphylla* and *Salsola laricifolia*, which were best modeled using crown projection volume, and *Caragana brachypoda*, which was best fitted using crown projection area, the BGB of the remaining 8 species was most accurately estimated as a function of AGB (Figure 3). The fitting equations for the 11 species are detailed in Tables A4 and A5.

Based on the optimal shrub biomass estimation models and relevant literature, the AGB, BGB, and total biomass of the 61 shrub communities in Western Inner Mongolia were calculated as 21.07 ± 21.22, 13.31 ± 17.22, and 34.39 ± 37.04 kg/100 m^2^, respectively (Table 1). The estimation equations for belowground and aboveground biomass of all xerophytic shrub species used in this study are provided in Table A6.

**TABLE 1:** Biomass of shrub communities in central and western Inner Mongolia.

Variable	Maximum	Minimum	Mean	Standard deviation
Aboveground biomass (kg/100 m^2^)	89.64	0.22	21.07	21.22
Belowground biomass (kg/100 m^2^)	103.77	0.15	13.31	17.22
Total biomass (kg/100 m^2^)	193.41	0.52	34.39	37.04

### 3.2 Key Environmental Factors Influencing Total Biomass and Species Richness in Arid Shrublands

**FIGURE 4:**
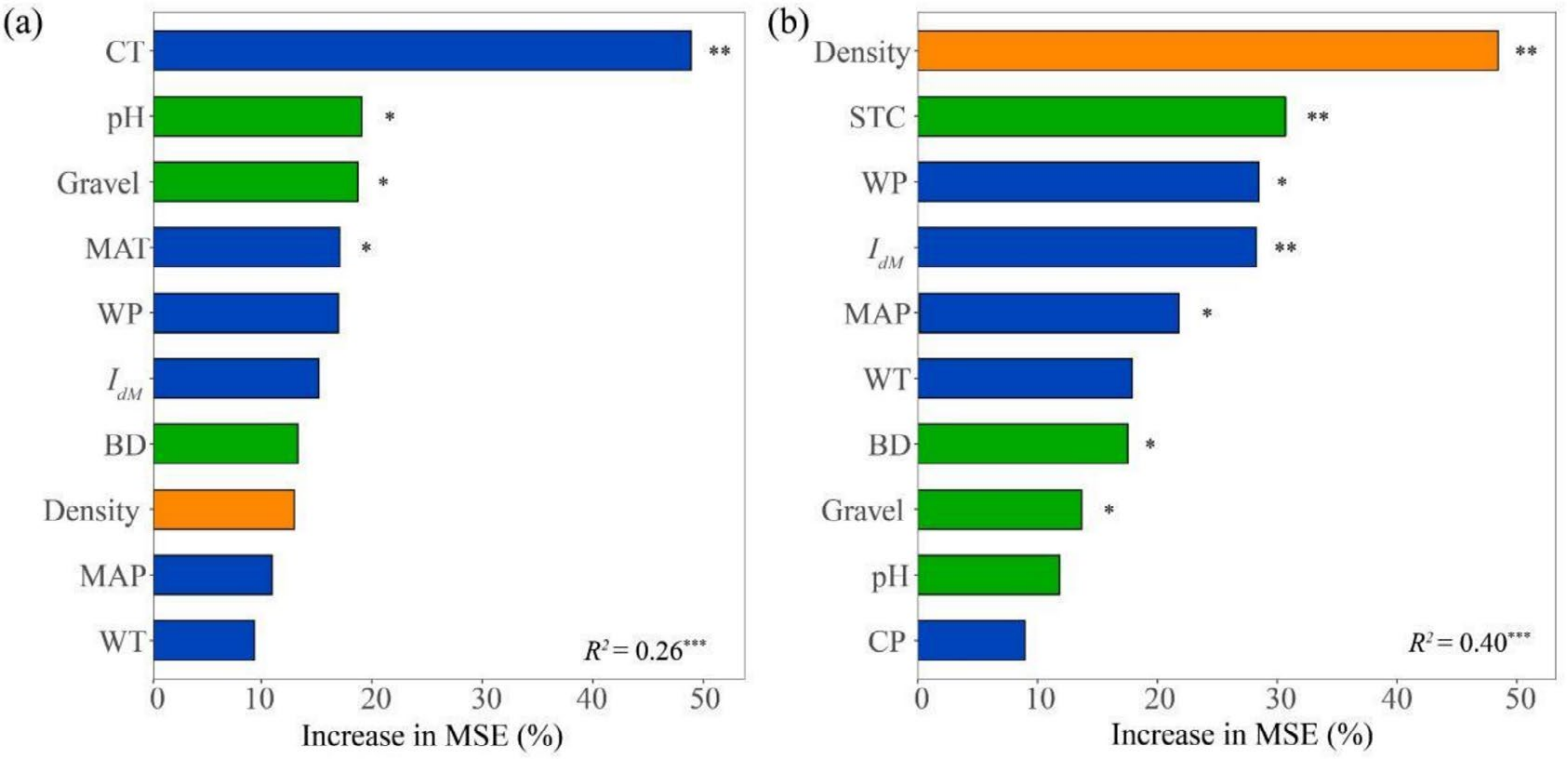
Random Forest analysis identifying key environmental factors influencing (a) total community biomass and (b) species richness (SR) in xerophytic shrub communities of western Inner Mongolia. *R* ^*2*^ indicates the percentage of variance explained by all 15 variables. Variables are ranked by their importance as measured by the percentage increase in mean squared error (%IncMSE) when the variable is randomly permuted. Bars represent the %IncMSE values. BD, Bulk density; CP, Precipitation of the coldest quarter; CT, Mean temperature of the coldest quarter; Density, Shrub density; Gravel, Soil‐to‐stone ratio; *I*_*dM*_, De Martonne index; MAP, Mean annual precipitation; MAT, Mean annual temperature; STC, Soil total carbon; WP, Precipitation of the warmest quarter; WT, Mean temperature of the warmest quarter; Significant levels: *p < 0.05; **p < 0.01 and ***p < 0.001.

Random forest models were used to evaluate and rank the environmental factors affecting total biomass and species richness in arid shrubland communities. The results indicated that environmental factors had a relatively low explanatory power for total shrub biomass, accounting for only 26% of the variance (cross‐validated *R* ^2^ = 0.26, *p* < 0.001). Among the factors considered, CT (%IncMSE = 48.66, *p* = 0.01) was identified as the most important influencing factor for total shrub biomass. This was followed by soil pH (%IncMSE = 18.85, *p* = 0.03) and the soil‐to‐stone ratio (Gravel, %IncMSE = 18.51, *p* = 0.03; Figure 4a).

For community species richness (SR), the random forest model explained 40% of the variance (cross‐validated *R* ^2^ = 0.40, *p* < 0.001). Seven environmental factors had significant effects on SR. Shrub density (%IncMSE = 48.44, *p* = 0.01) was the most significant influencing factor, followed by STC (%IncMSE = 30.8, *p* = 0.01). Among climatic factors, WP (%IncMSE = 28.47, *p* = 0.02), *I*_*dM*_ (%IncMSE = 28.25, *p* = 0.01), and MAP (%IncMSE = 21.67, *p* = 0.03) all significantly influenced SR (Figure 4b).

### 3.3 Influence of Environmental Factors on the Relationship Between Community Biomass and Species Richness

**FIGURE 5:**
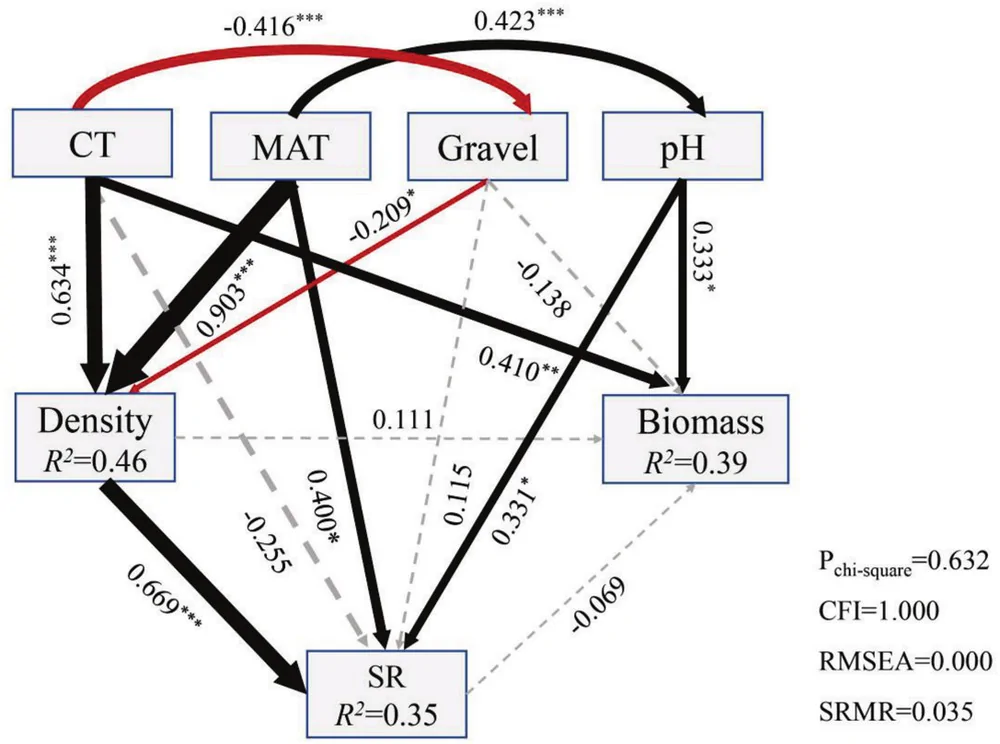
Structural Equation Model (SEM) depicting the direct and indirect effects of environmental factors on total community biomass in xerophytic shrub communities. Numbers are standardized path coefficients (*r*_*∂*_). *R* ^2^ denotes the proportion of variance in the dependent variable explained by the independent variables. Red and black arrows indicate positive and negative relationships, respectively; solid and dashed arrows represent significant and non‐significant paths, respectively. Biomass, Total biomass; CT, Mean temperature of the coldest quarter; Density, Shrub density; Gravel, Soil–stone ratio; MAT, Mean annual temperature; Model fit indices, Chi‐square *p* value, CFI (comparative fit index), RMSEA (root mean square error of approximation), SRMR (standardized root mean square residual); SR, Species richness; Significant levels: *p < 0.05 and ***p < 0.001.

**FIGURE 6:**
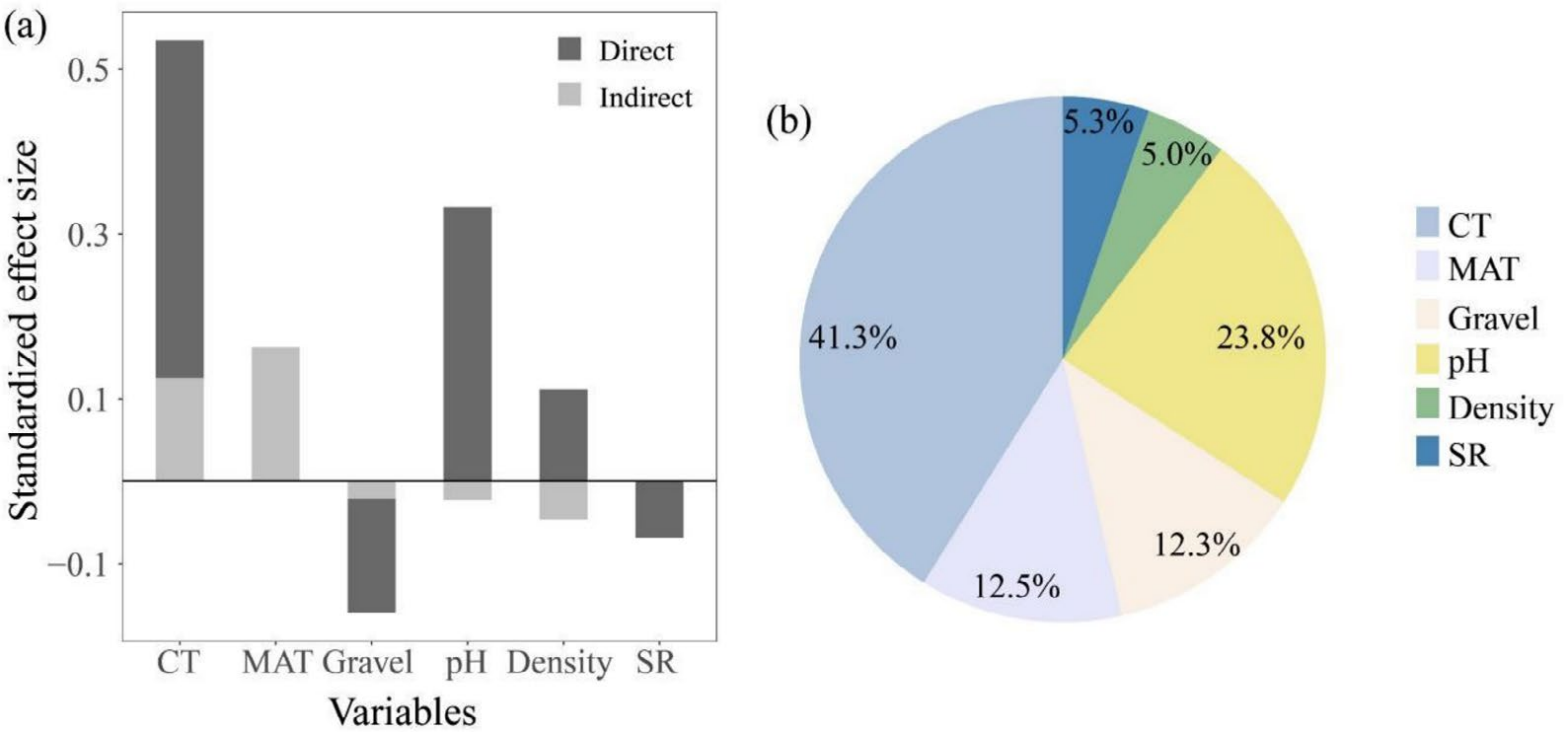
Relative contributions of environmental factors in the structural equation models. Bar plot (a) shows the total effects (direct + indirect) of each factor on total biomass. Pie chart (b) shows the relative contributions of predictors to the explained variance in total biomass. CT, Mean temperature of the coldest quarter; Density, Shrub density; Gravel, Soil–stone ratio; MAT, Mean annual temperature; SR, Species richness.

Structural Equation Modeling (SEM) was conducted using the environmental factors—mean CT, MAT, soil‐to‐stone ratio, and soil pH—identified by the random forest model as significantly affecting total community biomass. The model demonstrated a good fit to the data, as indicated by the following indices: a non‐significant chi‐square test (*p* = 0.632 > 0.05), RMSEA = 0.000 and SRMR = 0.035 (both < 0.05), and CFI = 1.000 (Figure 5). This model explained 39% (*R* ^2^ = 0.39) of the variance in total biomass. The results revealed that total shrub biomass was primarily influenced by positive direct effects from CT (path coefficient, *r*_*∂*_ = 0.410, *p* < 0.01) and soil pH (*r*_*∂*_ = 0.333, *p* < 0.01) (Figures 5 and 6a), with relative contributions of 41.3% and 23.8%, respectively (Figures 5, 6b). In contrast, the influence of MAT on total biomass was mainly indirect, accounting for 12.5% of the relative contribution (Figure 6). The soil‐to‐stone ratio exerted a non‐significant negative effect on community biomass (*p* > 0.05; Figures 5, 6a). Furthermore, neither community SR (*r*_*∂*_ = −0.069, *p* > 0.05) nor shrub density (*r*_*∂*_ = 0.111, *p* > 0.05) showed a significant direct relationship with total community biomass (Figure 5).

## 4 Discussion

### 4.1 Shrub Biomass Estimation

Among the optimal biomass estimation models for the 11 shrub and semi‐shrub species in western Inner Mongolia, the crown projection volume (hereafter referred to as volume) served as the best predictor variable for the AGB estimation models in the greatest number of species. This finding aligns with previous studies (Zhao et al. 2019). Plant volume is a relatively stable metric that better reflects the approximately columnar or conical architecture of shrubs, thereby supporting more accurate biomass estimation. However, the relationship between predictor variables and biomass is also closely linked to shrub life‐form and even to individual shrub density (Sheil and Bongers 2020). The adjusted coefficient of determination (*R* ^2^_adj_) for the best‐fitting biomass models was generally lower for succulent‐leaved plants such as *Salsola laricifolia*, *Salsola passerina*, and *Nitraria sphaerocarpa*. In contrast, model fits tended to be better for species with reduced leaves and more developed woody structures, such as *Caragana brachypoda* and *Oxytropis aciphylla*.

The high variability in community biomass observed in this study is attributed to multiple factors, resulting in a large standard deviation. First, shrub communities in this region are often characterized by a patchy distribution pattern, where dominant species such as *Nitraria tangutorum* or *Artemisia ordosica* form discrete clumps interspersed with bare ground, leading to high spatial variability in biomass at both the quadrat and site levels. Second, the age structure of shrub populations varies considerably among sites; older, established individuals can accumulate substantial biomass over decades, while younger stands contribute far less, further amplifying the among‐site variance. Third, as perennial plants, shrubs integrate growth responses over many years, and legacy effects of past climatic conditions may lead to persistent differences in biomass. These combined factors explain why the standard deviation of community biomass exceeds its mean value.

### 4.2 Influence of Environmental Factors on Biomass and Species Diversity in Arid Shrublands

The results reveal that the biomass of xerophytic shrubs is primarily influenced by temperature, particularly the mean temperature of the coldest season (CT). This contrasts with numerous studies identifying precipitation seasonality (Li et al. 2025) or water availability (Boutraa 2010) as the main limiting factors for plant growth in desert regions. Through long‐term evolution, xerophytic shrubs have developed a suite of strategies to cope with drought stress. Some species possess deeply lobed leaves with hairs and sunken stomata, or exhibit leaf reduction to minimize transpirational surface area. Others have developed succulent leaves with water‐storing tissues. Furthermore, most xerophytic shrubs form extensive root systems to maximize soil water uptake (Zhao and Yan 2008).

However, temperate xerophytic shrubs are also constrained by low temperatures, with CT emerging as the most significant factor affecting their biomass. Extreme minimum temperatures in desert regions can reach −36°C (Zhao and Yan 2008). Such winter cold extremes threaten plant overwintering and can impair growth in the following year. Indeed, extreme winter lows on the Mongolian Plateau have been shown to cause a significant decline in desert vegetation cover in the subsequent growing season (John et al. 2013).

This temperature sensitivity may also be linked to the origin and formation of the regional flora (Zheng et al. 2019). The contemporary plant community is a product of historical filtering. Many endemic species in the Alxa Desert exhibit ancient, tropical characteristics associated with the paleo‐Mediterranean flora (Zhao and Yan 2008). This flora was largely established during the Tertiary, with numerous ancient genera and species preserved and further developed through the Quaternary (Liu 1995). During the Paleogene, global temperatures were warmer than today, with hot summers and milder winters, and vegetation was dominated by subtropical xerophytes. Following major geological events and dramatic climate changes, including the retreat of the ancient Tethys Sea, the climate became drier and temperature patterns altered (Verdú et al. 2003), potentially establishing low temperatures as a key limiting factor for plant growth in the region. Consistent with this, several studies indicate that minimum temperature is an important driver of adaptive genetic variation in herbaceous (Johnson et al. 2012), shrub (Horning et al. 2010), and forest species (St Clair et al. 2005).

The mean temperature of the coldest season (CT) is the primary climatic driver of shrub biomass, which has significant implications for predicting the responses of xeric shrub ecosystems to ongoing climate change. Climate projections for the Mongolian Plateau and northern China indicate a continued warming trend, with winter temperatures expected to rise significantly (Jiang et al. 2016). On one hand, rising winter temperatures may alleviate cold stress for xerophytic shrubs, particularly for species with tropical or paleo‐Mediterranean origins that are sensitive to low‐temperature extremes. A global synthesis of shrub dynamics found that shrubs expanded at 84% of studied sites worldwide in response to climate warming, with winter temperature being a major contributor to shrubline shift rates (Wu et al. 2024). On the other hand, the response is unlikely to be uniform across species or communities. In our study region, different shrub species—such as the deep‐rooted *Nitraria tangutorum*, the drought‐tolerant *Artemisia ordosica*, and the succulent *Salsola passerina*—possess distinct physiological strategies and thermal niches, which may lead to divergent responses to winter warming. Species that are currently limited by cold stress may benefit most from warming winters, potentially altering community composition and competitive dynamics.

Indeed, studies in other shrubland systems have demonstrated that the relationship between biomass and climatic factors can be context‐dependent, with the relative importance of temperature versus water availability varying along environmental gradients (Guo et al. 2019). Future studies should employ larger sample sizes and broader climate gradients to clearly test whether the drivers of shrub biomass exhibit threshold or nonlinear responses to temperature changes, and to explore whether the temperature‐dependent patterns observed in other ecosystems also apply to the xeric shrub communities of the Mongolian Plateau.

Soil‐to‐stone ratio, soil pH, and mean annual temperature (MAT) are also important factors influencing shrub biomass. The soil‐to‐stone ratio exerts a primarily indirect, negative effect on community biomass, aligning with findings from Iran where shrub biomass decreased significantly with increasing gravel content (Sanaei et al. 2018). Soils with high gravel content offer less physical protection for organic matter and nutrients (Zinn et al. 2007), impairing root nutrient uptake and ultimately reducing biomass. Overcoming multiple coexisting stressors is critical for xerophytic shrub growth (Gorai et al. 2014), and temperature is a key physiological constraint. In arid and semi‐arid regions, when water is not limiting, plant growth is often temperature‐limited (Evans and Etherington 1990). Temperature controls seed germination by influencing processes such as cell membrane permeability and the activity of membrane‐bound and cytosolic enzymes (Bewley and Black 1994; Gorai et al. 2014). Furthermore, temperature regulates microbial decomposition, root respiration, and the diffusion of enzymes and substrates (Jassal et al. 2008; Wang et al. 2014), thereby affecting shrub community biomass.

Nevertheless, the overall explanatory power of the environmental factors for shrub biomass in this study was relatively low. Among climatic variables, aside from the CT, other factors ranked lower in importance within the random forest model. MAT and MAP may not be primary drivers of shrub biomass here. One possible reason is that the studied shrubs were concentrated in the Alxa Desert and Ordos sandy lands, where climatic variation among communities was minimal, failing to establish a strong environmental gradient. The effect of mean annual temperature was indirect and its contribution was relatively low (12.5%). This may be because mean annual temperature does not directly reflect the tissue temperatures that determine plant growth rates; plant traits can decouple tissue temperature from air temperature (Gates 1980), weakening the correlation with biomass. Research across biomes suggests that leaf temperatures remain relatively stable across regions, with branch physiology and morphology capable of elevating leaf temperature above air temperature at northern latitudes (Helliker and Richter 2008). Future studies should therefore consider using measured plant or air temperatures during key growth periods for biomass estimation (Michaletz et al. 2014).

Furthermore, MAP showed no significant effect on shrub biomass in this study, despite extensive evidence that terrestrial plant biomass or productivity generally increases with precipitation (Bai et al. 2008). While water availability is a key growth limiter (Huxman et al. 2004), total precipitation does not equate to plant‐available water (Renée Brooks et al. 2009). Available water is mediated by plant life form and habitat. In sandy shrublands, soils have lower evaporation and higher permeability, favoring moisture conservation, so plant‐available water can be relatively abundant even with low rainfall. Therefore, even with lower rainfall, plant‐available water resources can still be relatively abundant. Conversely, plants such as *Nitraria tangutorum* and *Kalidium foliatum* are often found in saline‐alkaline floodplain depressions where soil moisture is less dependent on direct precipitation.

Beyond abiotic factors, biological interactions also influence community biomass. Herbaceous plants growing beneath the shrub layer can affect shrub biomass, a process itself modulated by climate. Studies indicate that understory herbs can influence shrub seedling growth and survival. In years with average rainfall, taller shrubs may indirectly benefit seedlings by suppressing herb competition. In contrast, during dry summers, herbs may inhibit the growth of mature shrubs and seedling survival, thereby affecting community biomass (Cuesta et al. 2010). Other research in Mediterranean semi‐arid regions suggests that the herb layer's influence on shrubs is consistently facilitative and increases with environmental harshness (Maestre et al. 2003). These biotic interactions may also have contributed to the patterns observed in this study.

### 4.3 Relationship Between Species Richness and Biomass in Arid Shrublands

The absence of a significant relationship between species richness (SR) and community biomass in our study contrasts with findings from some other shrubland studies (Guo et al. 2019), but aligns with observations in alpine shrublands (Zhao and Yan 2008). This result suggests that in the harsh, low‐diversity xerophytic shrub ecosystems of western Inner Mongolia, community biomass is not primarily governed by the number of species present. Instead, our findings are consistent with the mass‐ratio hypothesis, which posits that ecosystem functioning is predominantly determined by the traits and abundance of the dominant species (Smith et al. 2020). The average SR in our communities was remarkably low (only 3 species per 100 m^2^), with many communities being monodominant (e.g., *Artemisia ordosica* in Ordos sandy lands). Under such conditions, the biomass contribution of a single dominant or foundation species far outweighs that of subordinate species, thus overriding any potential effect of species richness per se.

The relationship is further complicated by a range of biotic and abiotic factors. Shrub biomass in these systems represents an accumulation over multiple years, making it susceptible to the legacy effects of past climate, plant age (Gower et al. 1996), and phenological constraints (Comstock and Ehleringer 1986). Furthermore, interspecific interactions, which can be particularly intense in stressful environments (Butterfield 2009), likely modulate the SR‐biomass relationship. While facilitation may enhance productivity under extreme stress, competition for water and nutrients could also intensify, potentially masking any positive effect of diversity (Schenk and Mahall 2002). The sparse herbaceous layer, though often overlooked, may also play a role, as its facilitative or competitive effects on shrubs can vary with climatic conditions (Cuesta et al. 2010; Maestre et al. 2003). Therefore, the net effect of species richness on biomass is likely the outcome of these complexes, and sometimes opposing, interactions.

It is important to acknowledge that our study, based on observational data and structural equation modeling, is designed to identify key environmental drivers and correlational patterns. While we discuss plausible mechanisms such as the mass‐ratio effect and interspecific interactions, our analysis does not directly test the physiological or ecological processes underpinning these mechanisms. Future experimental studies, for instance manipulating species composition or neighbor interactions, are required to disentangle the direct and indirect causal pathways and to validate the hypotheses generated here. Our findings nonetheless provide critical baseline data and highlight the primary role of winter temperature and soil properties as key filters shaping the structure and function of these vulnerable ecosystems.

Moreover, the use of long‐term average climate data (1979–2013) from the CHELSA database in this study may not fully capture interannual climate variability affecting shrub growth, particularly since perennial shrubs can respond rapidly to climatic conditions during their establishment phase. Shrub biomass results from cumulative growth over years and may be influenced by climate conditions during previous growing seasons. Future studies that incorporate climate data at interannual resolution—such as data from weather stations or gridded datasets with annual time resolution—and apply dendrochronological methods could help more accurately disentangle the legacy effects of past climate on current biomass.

## 5 Conclusions

This study demonstrates that in the xerophytic shrub ecosystems of western Inner Mongolia, climatic factors, particularly the mean temperature of the coldest season, are primary drivers of community biomass, alongside soil pH and gravel content. Shrub community species richness, in contrast, is mainly governed by shrub density and soil carbon content. Critically, we find no significant direct relationship between species richness and community biomass. This pattern is consistent with the mass‐ratio hypothesis, suggesting that the functional dominance of a few key species, rather than overall diversity, determines community biomass in these low‐diversity systems. However, our analysis also indicates that this relationship is likely modulated by complex interspecific interactions and the historical legacies of climate, underscoring the need for further investigation.

As the dominant vegetation on the western Mongolian Plateau, shrubs play a central role in maintaining local ecosystem functions. Given the harsh environmental conditions, ecosystem fragility, and the high vulnerability—yet slow recovery—of vegetation in this region, future research should prioritize a deeper understanding of biodiversity and ecosystem functioning in desert shrub ecosystems.

## Author Contributions

**Zijing Li:** conceptualization (equal), formal analysis (equal), investigation (equal), writing – original draft (equal), writing – review and editing (equal). **Rui Jiao:** methodology (equal), writing – original draft (equal), writing – review and editing (equal). **Zhiyong Li:** data curation (equal), investigation (equal), project administration (equal), writing – review and editing (equal). **Cunzhu Liang:** data curation (equal), investigation (equal), project administration (equal). **Tiejun Liu:** funding acquisition (equal), supervision (equal). **Wentao Liang:** supervision (equal), writing – original draft (equal). **Zhenhua Han:** writing – review and editing (equal). **Kaixuan Li:** writing – review and editing (equal). **Yanfei Zhang:** writing – review and editing (equal). **Yaru Feng:** writing – review and editing (equal). **Guoyong Wang:** funding acquisition (equal).

## Funding

This work was supported by Natural Science Foundation of Inner Mongolia Autonomous Region (2024QN03024), IWHR Research & Development Support Program (MK2023J01); 2024 Ordos Industrial Innovation and Entrepreneurship Talent Team Program—Water Resources Digital Intelligence and Ecological Environment Damage Restoration Innovation Team and the Inner Mongolia Helan Mountain National Nature Reserve (Public Welfare Forest Area) Major Tree Species Conservation Technology Research Project (NMTX‐ZC‐2025‐06).

## Conflicts of Interest

The authors declare no conflicts of interest.

## Supporting information


**Table A1.** Descriptive statistics of environmental factors for shrub communities in western Inner Mongolia.
**Table A2**. Descriptive statistics for shrub communities in western Inner Mongolia.
**Table A3**. Basic information of shrub species.
**Table A4**. Aboveground biomass estimation models for Shrubs and Semi‐Shrubs.
**Table A5**. Belowground biomass estimation models for Shrubs and Semi‐Shrubs.
**Table A6**. Allometric models for estimating total (aboveground and belowground) biomass of Shrubs and Semi‐Shrubs.

**Data S1:** Supporting Information.

## Data Availability

The data that support the findings of this study are available in the Supporting Information for this article.
