# Supplementary material for: The Mean Temperature of the Coldest Season is the Main Factor Influencing the Relationship Between Biodiversity and Biomass in the Desert Shrubland of Western Inner Mongolia
Source: Ecol Evol. 2026 Sep 10;16(9):e74353. doi: 10.1002/ece3.74353 (PMC13561634; doi:10.1002/ece3.74353)
Supplement: Supplementary file 1 [file ECE3-16-e74353-s002.docx]

Table A.1 Descriptive statistics of environmental factors for shrub communities in western Inner Mongolia

|  | Variable | Maximum | Minimum | Mean | Standard deviation |
| --- | --- | --- | --- | --- | --- |
| Climate factor | MAP（mm） | 409.00 | 37.00 | 115.41 | 81.42 |
|  | MAT（℃） | 10.80 | 3.60 | 8.62 | 1.42 |
|  | de Martonne index | 28.97 | 1.86 | 6.98 | 4.67 |
|  | CT（℃） | -6.50 | -14.20 | -9.01 | 1.54 |
|  | WT（℃） | 27.80 | 19.7 | 24.42 | 2.00 |
|  | CP（mm） | 6.00 | 0.00 | 2.03 | 1.71 |
|  | WP（mm） | 247.00 | 25.00 | 71.51 | 45.11 |
| Soil factor | pH | 10.30 | 7.78 | 9.19 | 0.67 |
|  | Bulk density（g/cm^3^） | 1.84 | 1.21 | 1.52 | 0.12 |
|  | Gravel | 68.48 | 0.00 | 14.14 | 14.58 |
|  | STN（g/kg） | 1.07 | 0.07 | 0.24 | 0.15 |
|  | STC（g/kg） | 1.51 | 18.41 | 6.46 | 3.66 |
|  | STP（g/kg） | 0.13 | 0.83 | 0.30 | 0.12 |

Table A.2 Descriptive statistics for shrub communities in western Inner Mongolia

| Variable | Maximum | Minimum | Mean | Standard deviation |
| --- | --- | --- | --- | --- |
| Species richness (species/100 m^2^) | 9.43 | 1.00 | 3.04 | 1.83 |
| Shrub density | 2.44 | 0.031 | 0.57 | 0.64 |

Table A.3 Basic information of shrub species

| species | Hight (m) | Diameter (m) | Area (m^2^) | Volume (m^3^) | Number |
| --- | --- | --- | --- | --- | --- |
| *Artemisia sphaerocephala* | 0.08-1.06 | 0.10-2.05 | 0.007-3.250 | 0.001-3.120 | 28 |
| *Brachanthemum pulvinatum* | 0.03-0.45 | 0.05-1.17 | 0.002-0.978 | 0.00005-0.440 | 28 |
| *Calligonum mongolicum* | 0.10-0.86 | 0.22-3.99 | 0.036-12.361 | 0.006-5.192 | 16 |
| *Caragana brachypoda* | 0.05-0.32 | 0.08-0.55 | 0.004-0.232 | 0.0002-0.051 | 24 |
| *Convolvulus tragacanthoides* | 0.04-0.18 | 0.06-0.36 | 0.002-0.142 | 0.0001-0.047 | 29 |
| *Gymnocarpos przewalskii* | 0.12-0.72 | 0.15-1.20 | 0.016-1.130 | 0.002-0.769 | 29 |
| *Kalidium foliatum* | 0.01-0.90 | 0.04-1.40 | 0.0009-1.531 | 0.00004-1.286 | 28 |
| *Nitraria sphaerocarpa* | 0.01-4.10 | 0.03-3.515 | 0.0007-9.430 | 0.00002-6.16 | 25 |
| *Oxytropis aciphylla* | 0.01-0.44 | 0.04-2.15 | 0.0009-3.63 | 0.00003-0.402 | 47 |
| *Salsola laricifolia* | 0.04-0.46 | 0.12-0.93 | 0.011-0.667 | 0.0004-0.307 | 10 |
| *Salsola passerina* | 0.02-0.48 | 0.03-1.03 | 0.002-3.293 | 0.00006-0.943 | 27 |

Table A.4 Aboveground Biomass Estimation Models for Shrubs and Semi-Shrubs

| **Species** | **Function** | **Crown diameter (m)** | ***R^2^_adj_*** | **Crown area (m^2^)** | ***R^2^_adj_*** | **Crown volume (m^3^)** | ***R^2^_adj_*** |
| --- | --- | --- | --- | --- | --- | --- | --- |
| *Artemisia sphaerocephala* | *y= ax + b* | *y=1.1079x-0.6181* | 0.428 | *y=1.1104x-0.2929* | 0.577 | *y=1.5448x-0.1663* | 0.778* |
|  | *y= ax^b^* | *y=0.2803x^2.6735* | 0.657 | *y=0.3848x^1.2896* | 0.634 | *y=0.6336x^1.0146* | 0.720 |
| *Brachanthemum pulvinatum* | *y= ax + b* | *y=0.6670x-0.1055* | 0.623 | *y=1.2611x-0.0060* | 0.705 | *y=5.6499x+0.02860* | 0.743 |
|  | *y= ax^b^* | *y=0.5672x^1.5993* | 0.654 | *y=0.6603x^0.7754* | 0.650 | *y=0.5561x^0.6552* | 0.740 |
| *Calligonum mongolicum* | *y= ax + b* | *y=0.2715x-0.1895* | 0.326 | *y=0.1295x-0.0154* | 0.315 | *y=0.0388x+0.0684* | 0.135 |
|  | *y= ax^b^* | *y=0.0709x^2.2381* | 0.562 | *y=0.0963x^1.0866* | 0.560 | *y=0.0808x^0.6089* | 0.331 |
| *Caragana brachypoda* | *y= ax + b* | *y=0.5036x-0.1102* | 0.690 | *y=0.8497x-0.0264* | 0.817 | *y=2.0904x-0.0012* | 0.959 |
|  | *y= ax^b^* | *y=0.9462x^2.2934* | 0.820 | *y=1.3650x^1.1632* | 0.828 | *y=1.9065x^0.8128* | 0761 |
| *Convolvulus*  *tragacanthoides* | *y= ax + b* | *y=0.2445x-0.03512* | 0.632 | *y=0.4263x+0.00759* | 0.630 | *y=1.6274x+0.01657* | 0.699 |
|  | *y= ax^b^* | *y=0.2732x^1.7407* | 0.497 | *y=0.3269x^0.8435* | 0.488 | *y=0.6140x^0.6477* | 0.570 |
| *Gymnocarpos przewalskii* | *y= ax + b* | *y=0.4190x-0.1080* | 0.546 | *y=0.4833x+0.0017* | 0.552 | *y=1.1479x+0.0495* | 0.435 |
|  | *y= ax^b^* | *y=0.7227x^2.0701* | 0.656 | *y=0.9466x^1.0368* | 0.6555 | *y=1.8746x^0.7617* | 0.606 |
| *Kalidium foliatum* | *y= ax + b* | *y=1.7142x-0.6090* | 0.809 | *y=1.4089x-0.07135* | 0.920 | *y=1.9947x+0.06525* | 0.951 |
|  | *y= ax^b^* | *y=1.9350x^2.6670* | 0.870 | *y=1.4673x^1.3531* | 0.89 | *y=2.2817x^0.92256* | 0.942 |
| *Nitraria sphaerocarpa* | *y= ax + b* | *y=5.3431x-1.5161* | 0.385 | *y=6.7053x-0.2486* | 0.505 | *y=19.9199x+0.1926* | 0.586 |
|  | *y= ax^b^* | *y=3.4549x^1.8936* | 0.453 | *y=4.6599x^0.9738* | 0.463 | *y=10.1087x^0.7443* | 0.470 |
| *Oxytropis aciphylla* | *y= ax + b* | *y=0.6128x-0.1137* | 0.719 | *y=1.2025x-0.0236* | 0.815 | *y=5.56246x-0.0032* | 0.808 |
|  | *y= ax^b^* | *y=0.7503x^2.2525* | 0.820 | *y=0.9975x^1.1120* | 0.830 | *y=2.7406x^0.695* | 0.822 |
| *Salsola laricifolia* | *y= ax + b* | *y=0.2493x+0.0552* | 0.324 | *y=0.2124x+0.1414* | 0.251 | *y=0.2753x+0.1564* | 0.228 |
|  | *y= ax^b^* | *y=0.6466x^0.9325* | 0.289 | *y=0.7061x^0.4324* | 0.253 | *y=0.8102x^0.3613* | 0.294 |
| *Salsola passerina* | *y= ax + b* | *y=0.4818x-0.03001* | 0.399 | *y=0.6787x+0.0744* | 0.346 | *y=1.8861x+0.1046* | 0.364 |
|  | *y= ax^b^* | *y=0.9388x^2.1612* | 0.657 | *y=1.1385x^1.0289* | 0.630 | *y=2.1094x^0.7678* | 0.679 |

Note: *R^2^_adj_*: adjusted coefficient of determination; *: Although the equation has the highest *R^2^_adj_*, the residuals of the equation do not conform to the normal distribution, so the equation is not selected as the optimal model

Table A.5 Belowground Biomass Estimation Models for Shrubs and Semi-Shrubs

| **Species** | **Function** | **Crown diameter (m)** | ***R^2^_adj_*** | **Crown area(m^2^)** | ***R^2^_adj_*** | **Crown volume(m^3^)** | ***R^2^_adj_*** | **Aboveground dry matter (kg)** | ***R^2^_adj_*** |
| --- | --- | --- | --- | --- | --- | --- | --- | --- | --- |
| *Artemisia sphaerocephala* | *y= ax + b* | *y=0.2374x-0.1157* | 0.387 | *y=0.2162x-0.0366* | 0.424 | *y=0.2364x-0.0029* | 0.341 | *y=0.1614x+0.0267* | 0.489 |
|  | *y= ax^b^* | *y=0.0764x^2.4509* | 0.416 | *y=0.1018x^1.1783* | 0.398 | *y=0.1356x^0.8359* | 0.361 | *y=0.2456x^0.9174* | 0.641 |
| *Brachanthemum pulvinatum* | *y= ax + b* | *y=0.3348x-0.0468* | 0.342 | *y=0.5951x+0.0070* | 0.339 | *y=2.2470x+0.0303* | 0.244 | *y=0.4122x+0.0172* | 0.364 |
|  | *y= ax^b^* | *y=0.3179x^1.7608* | 0.449 | *y=0.3788x^0.8567* | 0.450 | *y=0.8898x^7197* | 0.57 | *y=0.5369x^1.0592* | 0.638 |
| *Calligonum mongolicum* | *y= ax + b* | *y=0.6948x-0.5422* | 0.299 | *y=0.3347x-0.1010* | 0.296 | *y=0.1024x+0.1109* | 0.134 | *y=2.5393x-0.05412* | 0.912 |
|  | *y= ax^b^* | *y=0.1009x^3.1597* | 0.553 | *y=0.1554x^1.5388* | 0.555 | *y=0.1197x^0.8850* | 0.349 | *y=3.3882x^1.3097* | 0.837 |
| *Caragana brachypoda* | *y= ax + b* | *y=0.4638x-0.1045* | 0.840 | *y=0.7653x-0.0256* | 0.948 | *y=1.6880x+0.0026* | 0.884 | *y=0.7662x+0.0060* | 0.826 |
|  | *y= ax^b^* | *y=1.0387x^2.5888* | 0.817 | *y=1.5489x^1.3076* | 0.817 | *y=1.1213x^0.9124* | 0.749 | *y=1.9654x^1.0689* | 0.888 |
| *Convolvulus tragacanthoides* | *y= ax + b* | — | — | — | — | *y=0.2198x+0.0104* | 0.007 | *y=0.2387x+0.001996* | 0.174 |
|  | *y= ax^b^* | *y=0.8433x^1.1307* | 0.062 | *y=0.0940x^0.5637* | 0.066 | *y=0.1505x^0.4938* | 0.118 | *y=0.1542x^0.9140* | 0.344 |
| *ymnocarpos przewalskii* | *y= ax + b* | *—* | — | *—* | — | *—* | — | *y=0.4720x-0.002032* | 0.213 |
|  | *y= ax^b^* | *y=0.2365x^2.0280* | 0.3 | *y=0.3091x^1.0181* | 0.301 | *y=0.5853x^0.7365* | 0.268 | *y=0.7034x^1.0216* | 0.511 |
| *Kalidium*  *foliatum* | *y= ax + b* | *y=0.6583x-0.1723* | 0.290 | *y=0.5337x+0.0364* | 0.324 | *y=0.8168x+0.0797* | 0.398 | *y=0.4287x+0.0464* | 0.464 |
|  | *y= ax^b^* | *y=3.0633x^3.0588* | 0.592 | *y=1.0997x^1.5774* | 0.627 | *y=1.1411x^1.1475* | 0.761 | *y=0.7896x^1.2255* | 0.784 |
| *Nitraria sphaerocarpa* | *y= ax + b* | *y=2.7003x-0.3450* | 0.186 | *y=3.3180x+0.3124* | 0.239 | *y=8.8468x+0.5896* | 0.219 | *y=0.5281x+0.3905* | 0.567 |
|  | *y= ax^b^* | *y=2.2351x^1.6628* | 0.176 | *y=2.9627x^0.8737* | 0.191 | *y=4.3662x^0.5691* | 0.129 | *y=1.4894x^0.8930* | 0.434 |
| *Oxytropis aciphylla* | *y= ax + b* | *y=0.1077x-0.0175* | 0.659 | *y=0.2062x-0.0013* | 0.711 | *y=0.9992x+0.0018* | 0.758 | *y=0.1525x+0.0040* | 0.685 |
|  | *y= ax^b^* | *y=0.1022x^1.8545* | 0.585 | *y=0.1331x^0.9257* | 0.606 | *y=0.2994x^0.7176* | 0.589 | *y=0.1213x^0.8033* | 0.679 |
| *Salsola laricifolia* | *y= ax + b* | *y=0.4087x-0.0008* | 0.538 | *y=0.3954x+0.1281* | 0.592 | *y=0.5387x+0.1524* | 0.618 | *—* | — |
|  | *y= ax^b^* | *y=0.7556x^0.9263* | 0.32 | *y=0.8626x^0.4597* | 0.34 | *y=0.9549x^0.3635* | 0.337 | *—* | — |
| *Salsola passerina* | *y= ax + b* | *y=0.0944x+0.0016* | 0.168 | *y=0.1264x+0.023* | 0.125 | *y=0.3261x+0.0299* | 0.109 | *y=0.1374x+0.0184* | 0.203 |
|  | *y= ax^b^* | *y=0.1407x^1.6869* | 0.349 | *y=0.1676x^0.8155* | 0.345 | *y=0.2611x^0.5953* | 0.356 | *y=0.1360x^0.7362* | 0.476 |

Note: *R^2^_adj_*: adjusted coefficient of determination.

Table A.6 Allometric Models for Estimating Total (Aboveground and Belowground) Biomass of Shrubs and Semi-Shrubs

| Species | Biomass | Function | Variable | *R^2^* |  | Model parameter | | |  |  | Units of variables | | | | | |  |
| --- | --- | --- | --- | --- | --- | --- | --- | --- | --- | --- | --- | --- | --- | --- | --- | --- | --- |
|  |  |  |  |  | *a* | | *b* | *c* | | *H* | | *D* | *A* | *V* | *M_a_* | *M* | |
| *Ammopiptanthus mongolicus* | AGB | *M = aA^b^* | *A* | 0.92 | 721.94 | | 1.00 |  | |  | |  | m^2^ |  |  | g | |
|  | BGB | *M = aA^b^* | *A* | 0.86 | 362.17 | | 1.11 |  | |  | |  | m^2^ |  |  | g | |
| *Amygdalus mongolica* | AGB | *M* = *a×V+b* | V | 0.83 | 0.9781 | | 0.0201 |  | |  | |  |  | m^3^ |  | kg | |
|  | BGB | *M = aM_a_^b^* | *M_a_* | 0.71 | 0.5357 | | 0.0071 |  | |  | |  |  |  | kg | kg | |
| *Ajania fruticulosa* | TB | *M* = *aD^b^* | *D* | 0.79 | 17.5388 | | 1.596 |  | |  | | cm |  |  |  | g | |
| *rtemisia ordosica* | AGB | *M = aA^b^* | *A* | 0.81 | 1.154 | | 1.257 |  | |  | |  | m^2^ |  |  | kg | |
|  | BGB | *M = aA^b^* | *A* | 0.66 | 0.28 | | 1.036 |  | |  | |  | m^2^ |  |  | kg | |
| *Artemisia sphaerocephala* | AGB | *M = aV^b^* | *V* | 0.72 | 0.6336 | | 1.0146 |  | |  | |  |  | m^3^ |  | kg | |
|  | BGB | *M = aM_a_^b^* | *M_a_* | 0.64 | 0.2456 | | 0.9174 |  | |  | |  |  |  | kg | kg | |
| *Brachanthemum pulvinatum* | AGB | *M = a×V+b* | *V* | 0.74 | 5.6499 | | 0.02860 |  | |  | |  |  | m^3^ |  | kg | |
|  | BGB | *M = aM_a_ ^b^* | *M_a_* | 0.64 | 0.5369 | | 1.0592 |  | |  | |  |  |  | kg | kg | |
| *Calligonum mongolicum* | AGB | *M = aA^b^* | *A* | 0.56 | 0.0963 | | 1.0866 |  | |  | |  | m^2^ |  |  | kg | |
|  | BGB | *M = a×M_a_+ b* | *M_a_* | 0.91 | 2.5393 | | -0.0541 |  | |  | |  |  |  | kg | kg | |
| *Caragana brachypoda* | AGB | *M = a×V+b* | *V* | 0.96 | 2.0904 | | -0.0012 |  | |  | |  |  | m^3^ |  | kg | |
|  | BGB | *M = a×A+b* | *A* | 0.95 | 0.7653 | | -0.0256 |  | |  | |  | m^2^ |  |  | kg | |
| *Caragana intermedia* | AGB | *M = a×e^b×H^* | *H* | 0.78 | 0.052 | | 3.054 |  | | m | |  |  |  |  | kg | |
|  | BGB | *M = aH^b^* | *H* | 0.74 | 1.302 | | 2.368 |  | | m | |  |  |  |  | kg | |
| *aragana korshinskii* | AGB | *M = aV^b^* | *V* | 0.75 | 467.24 | | 0.79 |  | |  | |  |  | m^3^ |  | g | |
|  | BGB | *M = aV^b^* | *V* | 0.52 | 499.85 | | 0.97 |  | |  | |  |  | m^3^ |  | g | |
| *Caragana microphylla* | AGB | *M* = *a×V+b* | *V* | 0.92 | 842.5 | | 16.82 |  | |  | |  |  | m^3^ |  | g | |
|  | BGB | *M* = *a×V+b* | *V* | 0.86 | 1477.25 | | 100.54 |  | |  | |  |  | m^3^ |  | g | |
| *Caragana stenophylla* | AGB | *M = aA^b^* | *A* | 0.55 | 159.39 | | 1.07 |  | |  | |  | m^2^ |  |  | g | |
|  | BGB | *M = aA^b^* | *A* | 0.40 | 126.34 | | 0.81 |  | |  | |  | m^2^ |  |  | g | |
| *Ceratoides latens* | AGB | *M = aV^b^* | *V* | 0.61 | 1.2331 | | 0.8691 |  | |  | |  |  | m^3^ |  | kg | |
|  | BGB | *M = a×M_a_+ b* | *M_a_* | 0.29 | 0.4373 | | 0.0232 |  | |  | |  |  |  | kg | kg | |
| *onvolvulus tragacanthoides* | AGB | *M = a×V+b* | *V* | 0.70 | 1.6274 | | 0.0166 |  | |  | |  |  | m^3^ |  | kg | |
|  | BGB | *M = aM_a_ ^b^* | *M_a_* | 0.34 | 0.1542 | | 0.9140 |  | |  | |  |  |  | kg | kg | |
| *phedra przewalskii* | AGB | *M =ax_1_+bx_2_ +c* | *x_1_=H*, *x_2_=A* | 0.84 | 22.64 | | 823.21 | 5.52 | | m | |  | m^2^ |  |  | g | |
|  | BGB | *M =ax_1_+bx_2_ +c* | *x_1_=H*, *x_2_=A* | 0.86 | 76.4 | | 898.28 | 13.31 | | m | |  | m^2^ |  |  | g | |
| *Gymnocarpos przewalskii* | AGB | *M* = a*D*^b^ | *D* | 0.66 | 0.7227 | | 2.0701 |  | |  | | m |  |  |  | kg | |
|  | BGB | *M = aM_a_^b^* | *M_a_* | 0.51 | 0.7034 | | 1.0216 |  | |  | |  |  |  | kg | kg | |
| *alidium foliatum* | AGB | *M* = *a×V+b* | *V* | 0.95 | 1.9947 | | 0.0653 |  | |  | |  |  | m^3^ |  | kg | |
|  | BGB | *M = aM_a_^b^* | *M_a_* | 0.78 | 0.3948 | | 1.2255 |  | |  | |  |  |  | kg | kg | |
| *Haloxylon ammodendron* | AGB | *M = aA^b^* | *A* | 0.82 | 1742.76 | | 1.29 |  | |  | |  | m^2^ |  |  | g | |
|  | BGB | *M = aA^b^* | *A* | 0.87 | 1465.17 | | 1.40 |  | |  | |  | m^2^ |  |  | g | |
| *Hedysarum fruticosum* var. *laeve* | TB | *M* = *a×V+b* | *V* | 0.88 | 0.2251 | | 2829.91 |  | |  | |  |  | cm^3^ |  | g | |
| *Helianthemum songaricum* | AGB | *M = a(AH)^b^* | *A*, *H* | 0.956 | 1.23 | | 2.01 |  | | cm | |  | m^2^ |  |  | g | |
|  | BGB | *M =a(AH)^2^+b(AH)+c* | *A*, *H* | 0.89 | 0.52 | | 4.43 | 17.79 | | cm | |  | m^2^ |  |  | g | |
| *Nitraria sphaerocarpa* | AGB | *M* = *a×V+b* | *V* | 0.59 | 19.9199 | | 0.1926 |  | |  | |  |  | m^3^ |  | kg | |
|  | BGB | *M = a×M_a_+ b* | *M_a_* | 0.57 | 0.5281 | | 0.3905 |  | |  | |  |  |  | kg | kg | |
| *Nitraria tangutorum* | AGB | *M = a×V+b* | *V* | 0.92 | 273.10 | | 92.99 |  | |  | |  |  | m^3^ |  | g | |
|  | BGB | *M = a×V+b* | *V* | 0.88 | 492.64 | | 185.27 |  | |  | |  |  | m^3^ |  | g | |
| *Oxytropis aciphylla* | AGB | *M = aA^b^* | *A* | 0.83 | 0.9975 | | 1.1120 |  | |  | |  | m^2^ |  |  | kg | |
|  | BGB | *M* = *a×V+b* | *V* | 0.77 | 0.9992 | | 0.0018 |  | |  | |  |  | m^3^ |  | kg | |
| *Potaninia mongolica* | AGB | *M* = *a×V+b* | *V* | 0.85 | 1.3175 | | 0.0041 |  | |  | |  |  | m^3^ |  | kg | |
|  | BGB | *M = aM_a_^b^* | *M_a_* | 0.77 | 0.1607 | | 0.6039 |  | |  | |  |  |  | kg | kg | |
| *eaumuria soongarica* | AGB | *M* = *a×V+b* | *V* | 0.73 | 1340.68 | | 19.76 |  | |  | |  |  | m^3^ |  | g | |
|  | BGB | *M = aA^b^* | A | 0.42 | 484.77 | | 1.04 |  | |  | |  | m^2^ |  |  | g | |
| *alix psammophila* | AGB | *M = aV^b^* | *V* | 0.66 | 261.44 | | 0.73 |  | |  | |  |  | m^3^ |  | g | |
|  | BGB | *M = aV^b^* | *V* | 0.62 | 107.63 | | 0.70 |  | |  | |  |  | m^3^ |  | g | |
| *Salsola laricifolia* | AGB | *M* = *a×D+b* | *D* | 0.32 | 0.2493 | | 0.0552 |  | |  | | m |  |  |  | kg | |
|  | BGB | *M* = *a×V+b* | *V* | 0.62 | 0.5387 | | 0.1524 |  | |  | |  |  | m^3^ |  | kg | |
| *Salsola passerina* | AGB | *M = aV^b^* | *V* | 0.68 | 2.1094 | | 0.7678 |  | |  | |  |  | m^3^ |  | kg | |
|  | BGB | *M = aM_a_^b^* | *M_a_* | 0.48 | 0.1360 | | 0.7362 |  | |  | |  |  |  | kg | kg | |
| *arcozygium xanthoxylon* | AGB | *M = aV^b^* | *V* | 0.84 | 1260.34 | | 0.98 |  | |  | |  |  | m^3^ |  | g | |
|  | BGB | *M = aV^b^* | *V* | 0.74 | 512.97 | | 1.01 |  | |  | |  |  | m^3^ |  | g | |
| *Sympegma regelii* | AGB | *M = a×V+b* | *V* | 0.89 | 2.7146 | | 0.0210 |  | |  | |  |  | m^3^ |  | kg | |
|  | BGB | *M = aM_a_^b^* | *M_a_* | 0.72 | 0.5902 | | 0.9563 |  | |  | |  |  |  | kg | kg | |
| *Tamarix chinensis* | AGB | *M = aA^b^* | *A* | 0.999 | 0.0048 | | 1.3745 |  | |  | |  | cm^2^ |  |  | g | |
|  | BGB | *M = aA^b^* | *A* | 0.98 | 1222.6 | | 1.0081 |  | |  | |  | cm^2^ |  |  | g | |

Note: AGB: aboveground biomass; BGB: belowground biomass; TB: total biomass; *H*: height; *D*: mean crown diameter; *A*: crown projection area; *V*: crown projection volume; *M_a_*: aboveground dry mass; *M*: corresponding organ dry mass; *R^2^*: coefficient of determination.
